# Exploratory Study Identifies Matrix Metalloproteinase-14 and -9 as Potential Biomarkers of Regorafenib Efficacy in Metastatic Colorectal Cancer

**DOI:** 10.3390/cancers16162855

**Published:** 2024-08-15

**Authors:** Mitsukuni Suenaga, Tetsuo Mashima, Naomi Kawata, Shingo Dan, Hiroyuki Seimiya, Kensei Yamaguchi

**Affiliations:** 1 Gastroenterology Center, Cancer Institute Hospital of Japanese Foundation for Cancer Research, 3-8-31 Ariake, Koto-ku, Tokyo 135-8550, Japan; naomi.kawata@jfcr.or.jp (N.K.); kensei.yamaguchi@jfcr.or.jp (K.Y.); 2 Department of Clinical Oncology, Tokyo Medical and Dental University (TMDU), 1-5-45 Yushima, Bunkyo-ku, Tokyo 113-8519, Japan; 3 Division of Molecular Biotherapy, Cancer Chemotherapy Center, Japanese Foundation for Cancer Research, 3-8-31 Ariake, Koto-ku, Tokyo 135-8550, Japan; tmashima@jfcr.or.jp (T.M.); hseimiya@jfcr.or.jp (H.S.); 4 Division of Molecular Pharmacology, Cancer Chemotherapy Center, Japanese Foundation for Cancer Research, 3-8-31 Ariake, Koto-ku, Tokyo 135-8550, Japan; sdan@jfcr.or.jp

**Keywords:** MMPs, regorafenib, metastatic colorectal cancer, biomarker

## Abstract

**Simple Summary:**

Regorafenib offers longer survival for patients with refractory metastatic colorectal cancer (mCRC). We aimed to identify biomarkers for regorafenib through preclinical and translational studies. In silico analysis identified matrix metalloproteinase (MMP)-14 and MMP-9 as key biomarkers. Validation in patients receiving regorafenib or FTD/TPI showed that high MMP-14 levels were correlated with a better response to regorafenib. Additionally, lower MMP-9 levels before the second cycle were linked to improved disease control and survival. These findings suggest that MMP-14 and MMP-9 could serve as prognostic markers for regorafenib efficacy.

**Abstract:**

In identifying biomarkers for anticancer drugs, the lack of objectivity in selecting candidate factors makes interpretation difficult. We performed preclinical analysis and a translational validation study to identify candidate biomarkers for regorafenib efficacy in metastatic colorectal cancer (mCRC). Using in silico COMPARE analysis with a human cancer cell line panel, JFCR39, we selected candidate biomarkers whose expression correlates with regorafenib sensitivity. We validated predictive values in mCRC patients receiving regorafenib (discovery, *n* = 53) and FTD/TPI (control, *n* = 16). Blood samples were obtained at baseline (BL), before the second cycle (2nd), and at progressive disease (PD), and biomarker levels were measured using ELISA. Our analysis showed that high matrix metalloproteinase (MMP)-14 expression was associated with a high sensitivity to regorafenib. In the discovery cohort, high MMP-14 levels at BL and PD were correlated with tumor shrinkage and longer progression-free survival (PFS). A subsequent analysis of other related factors further indicated that the patients with decreased MMP-9 levels at the 2nd had higher disease control rates, tumor shrinkage, longer PFS, and overall survival than those with increased changes. These findings were not observed in the control cohort. Our study suggests MMP-14 and MMP-9 may serve as prognostic markers for regorafenib and provide insights into novel combination therapies with anti-MMP-9 agents or FTD/TPI.

## 1. Introduction

Regorafenib, an oral multi-kinase inhibitor, confers the benefit of longer survival in patients with refractory metastatic colorectal cancer (mCRC) according to the results from two phase 3 trials [1,2]. A retrospective exploratory study of the CORRECT trial showed potential prognostic effects of circulating DNA concentrations in patients receiving regorafenib, and tumor genotyping using circulating DNA were also assessed at the time of treatment, suggesting BEAMing, a technique based on emulsion PCR, analysis of circulating DNA as a viable approach to obtain real-time tumor-associated genotypic information in a noninvasive way [3].

Matrix metalloproteinases (MMPs) are proteolytic enzymes for extracellular matrix (ECM) and are involved in the regulation of tumor environments including angiogenesis, cell proliferation, apoptosis, migration, invasion, metastasis, and immunity [4,5]. Two types of MMPs are known: membrane-anchored and secreted-type MMPs. Of membrane-type (MT)-MMPs, MT1-MMP (MMP-14) activates MMP-2 by composing an MT1-MMP–tissue inhibitor of metalloproteinase (TIMP)-2–MMP-2 complex on the cell surface, leading to cell migration and angiogenesis [6,7]. In contrast, MT1-MMP activity is inhibited by TIMP-2 and MT1-MMP itself [8]. An adhesion molecule CD44 recruits its substrates, including MMP-9, MMP-2, and MT1-MMP, and it is also cleaved by MT1-MMP [9]. MT1-MMP activates MMP-9 bound to CD44, which activates TGF-β and releases VEGF trapped in the ECM accelerating tumor-related angiogenesis [10] (Supplementary Figure S1). In recent studies, MMP-2 and MMP-9 were reported to be a potential predictive or prognostic marker of anti-angiogenic therapy using bevacizumab: high-MMP-2 and low-MMP-9 plasma levels were correlated with better tumor response and better survival in recurrent high-grade glioma, and with better survival in HER-2-positive inflammatory breast cancer receiving neoadjuvant therapy [11]. In contrast, TIMPs inhibit the activity of various MMPs, including MMP-9. By suppressing MMP activity, TIMPs help prevent tissue destruction and contribute to the maintenance of a normal extracellular matrix [12].

Given the critical roles of MMPs and TIMPs in cancer biology, we hypothesized that MMP-9, MMP-14, TIMP-1, and MMP-2 could serve as prognostic biomarkers for regorafenib efficacy in mCRC patients. We performed preclinical in silico and in vitro analyses and a translational validation study to identify candidate biomarkers for regorafenib efficacy in patients with mCRC. The markers identified using these methods, which utilize databases from basic research, allow for a more scientific and logical analysis compared to retrospective studies. This process could also help uncover new drug mechanisms and discover new drug targets.

## 2. Materials and Methods

### 2.1. Study Design and Patients

**Figure 1:**
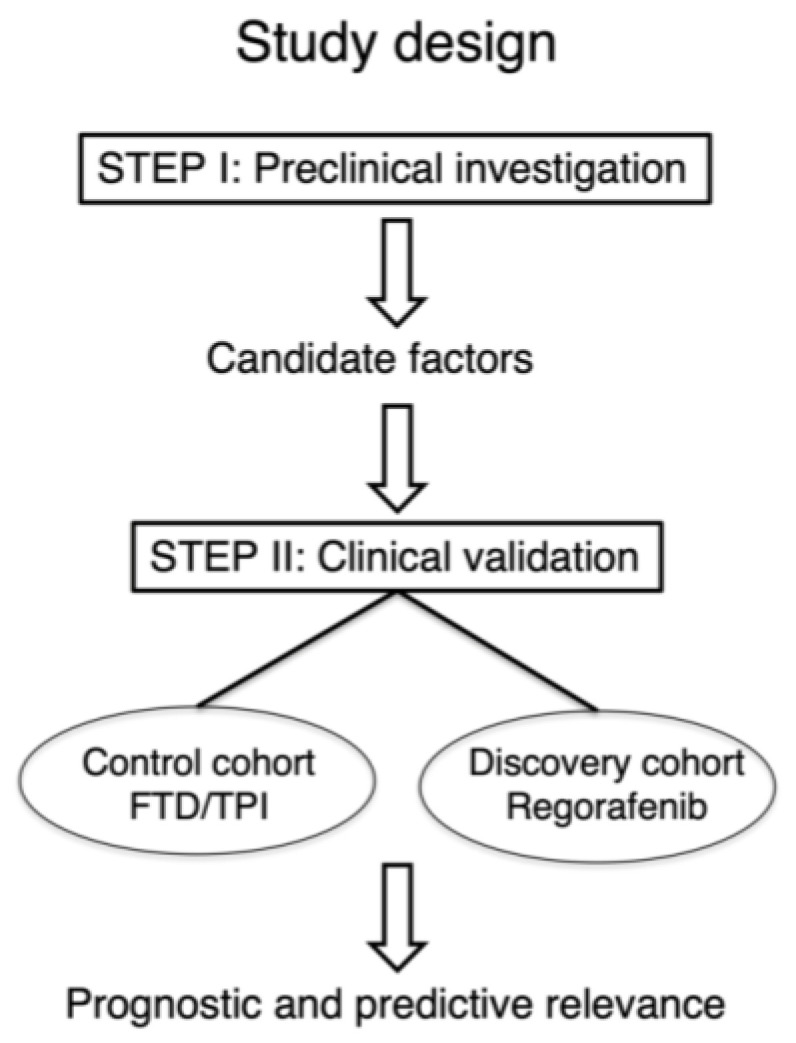
Study design. FTD/TPI, trifluridine/tipiracil.

This study consists of two steps as follows (Figure 1).

#### 2.1.1. STEP I: Preclinical Investigation

(i) Search for candidate factors related to regorafenib sensitivity based on our in-silico analysis. JFCR39 is a panel of 39 human cancer cell lines, with a drug sensitivity and gene expression database [13]. We obtained the sensitivity patterns (called ‘fingerprints’) of a variety of drugs and compounds, including regorafenib, in the 39 cell lines together with the gene expression data of the cell lines. Using COMPARE analysis, we extracted candidate factors whose expression showed an association with a high sensitivity to regorafenib, as described previously [13].

(ii) Evaluation of the relationship between protein changes and regorafenib sensitivity in colon cancer cell lines. To validate the relationship between the expression changes and regorafenib sensitivity, we first analyzed the sensitivity of human colon cancer cell lines (HT29 and HCT15) to regorafenib. Cells were maintained in RPMI-1640 medium (Life Technologies, Carlsbad, CA, USA) supplemented with 10% fetal bovine serum. Cells were treated with regorafenib at the indicated concentrations for 72 h. The cell number was measured using the CellTiter 96 AQueous One Solution Cell Proliferation Assay Kit (Promega, Madison, WI, USA). Secreted MMP-14 levels and their changes after regorafenib treatment (72 h) in human colon cancer cell lines were measured at the indicated concentrations. Secreted biomarker concentration in the cell culture medium was measured using Quantikine ELISA kits (R&D Systems, Minneapolis, MN, USA). Each experiment was performed at least twice with three replicates, and reproducible results were obtained.

#### 2.1.2. STEP II: Clinical Validation

(i) Patients with mCRC receiving regorafenib (discovery cohort, *n* = 53) and FTD/TPI (control, *n* = 16) were enrolled in this study.

(ii) Serum was separated from collected blood sample and stored at −80 °C.

(iii) Candidate biomarkers (MMP-2, MMP-9, MMP-14, TIMP-1) were measured using Quantikine ELISA kits (R&D Systems) according to the manufacturer’s instructions. The change patterns on treatment were categorized as ‘increased’ or ‘decreased’ from baseline (BL).

(iv) Tumor response was assessed according to RECIST 1.1. Progression-free survival (PFS) and overall survival (OS) were analyzed using Kaplan–Meier curves and log-rank test.

All patients met the eligibility criteria: history of previous standard chemotherapy including 5-fluorouracil, oxaliplatin, irinotecan, bevacizumab, and cetuximab or panitumumab for *KRAS* or *RAS* wild-type; measurable or evaluable disease according to the Response Evaluation Criteria in Solid Tumors (RECIST) 1.1; and signed informed consent. Adverse events were graded according to the Common Terminology Criteria for Adverse Events, version 4.0. Patients received 160 mg regorafenib (Bayer, Leverkusen, Germany) once daily from day 1 to day 21 every 4 weeks or FTD/TPI (Taiho Pharmaceutical Co., Ltd., Tokyo, Japan) at 35 mg/m^2^ two times daily for days 1–5 and 8–12, every 4 weeks. Doses were adjusted based on adverse events at a physician’s discretion, following the manufacturer’s recommendations. The study was approved by the Institutional Review Board and conducted at the Cancer Institute Hospital in accordance with the Declaration of Helsinki.

### 2.2. Analysis of Serum Factor Levels

Blood samples were obtained from 69 patients at baseline (BL) before the first dose of regorafenib or FTD/TPI, before the second cycle (2nd), and at progressive disease (PD). Separated serum was stored at −80 °C. The levels of serum factors were measured using Quantikine ELISA kits (R&D Systems). Serum MMP-14 and related proteins, MMP-2, MMP-9, and TIMP-1 levels were tested for investigation of the MMP signaling network.

### 2.3. Statistical Analysis

The primary endpoint of the study was progression-free survival (PFS), and the secondary endpoints were overall survival (OS), disease control rate (DCR), and tumor shrinkage (TS). PFS was defined as the interval between the date of starting treatment and the date of confirmed disease progression or death. The data of patients without disease progression or death were censored on the date of the last follow-up. OS was calculated from the date of starting treatment until the date of death from any cause. In patients who discontinued follow-up, data were censored on the date of the last follow-up. DCR was defined as the proportion of patients who achieved a complete response (CR), partial response (PR), or stable disease (SD). Chi-square tests were used to examine the differences in baseline patient characteristics between two cohorts. The tumor shrinkage was defined as a reduction of the sum of the longest diameters of a target by 0% or more when compared with BL. Fisher’s exact test was used to examine the associations between factor levels at any points, or between changes in factor levels and DCR or TS. Differences in serum factor levels at any points and level changes between BL and before the second cycle or PD were analyzed using Student’s *t*-test for the mean values or the Mann–Whitney U-test for the median values. Spearman’s rank correlation coefficient was calculated for correlations among serum factors, and Pearson’s correlation coefficient was also used for data with normal distribution. All analyses were performed using SPSS software (version 29.0; IBM Corporation, Armonk, NY, USA), and *p* < 0.05 was considered statistically significant.

## 3. Results

### 3.1. Preclinical Data Analysis

**Figure 2:**
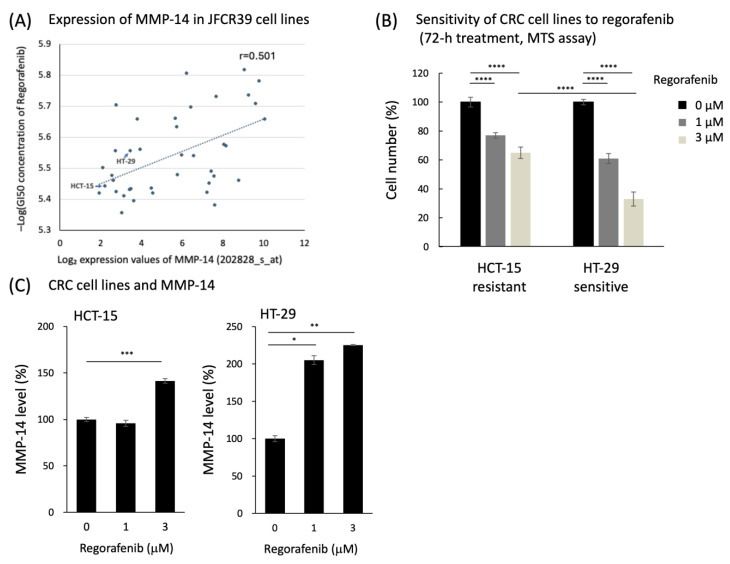
Preclinical data analysis. (**A**) Expression of MMP-14 in JFCR39 cell lines, (**B**) sensitivity of CRC cell lines to regorafenib, (**C**) human colon cancer cell line and secreted MMP-14 concentration after regorafenib treatment. Basal MMP-14 levels were 109 pg/mL and 268 pg/mL in the conditioned mediums of the two CRC cells, respectively. Statistical significance was evaluated by ANOVA followed by Tukey’s post hoc test using GraphPad Prism (version 8). * *p* < 0.01, ** *p* < 0.005, *** *p* < 0.001, **** *p* < 0.0001.

To search for candidate factors related to regorafenib sensitivity, we first performed an in silico COMPARE analysis with JFCR39, a panel of 39 human cell lines with an integrated database of multi-omics data and drug sensitivity data [13]. Using the analysis, we found an association between high expression of matrix metalloproteinase-14 (MMP-14) and high sensitivity to regorafenib (Figure 2A). Expression levels of MMP-14 (probe set 202828_s_at) were measured using the Affymetrix^®^ GeneChip^®^ Human Genome U133 Plus 2.0. The expression values were normalized across the JFCR39 cell lines using the MAS5 algorithm. To further test this relationship, we examined the expression of MMP-14 in human cancer cell lines with differential regorafenib sensitivity. In the analysis of sensitivity of CRC cell lines to regorafenib, HT-29 appeared to be sensitive while HCT-15 was shown as resistant (Figure 2B). Secreted MMP-14 levels and their changes after regorafenib treatment in human colon cancer cell line were assessed, in which the secreted MMP-14 concentration was normalized by cell number and indicated. MMP-14 levels were increased after regorafenib treatment in regorafenib-sensitive HT-29 cells, whereas no significant change was observed in regorafenib-resistant HCT-15 cells (Figure 2C).

### 3.2. Patient and Tumor BL Characteristics

The baseline characteristics are summarized in Table 1. Compared with the control cohort, the discovery cohort contained a higher percentage of male patients, patients with right-sided tumors, and patients with lung metastasis. The median PFS and OS were 2.7 and 8.1 months in the discovery cohort, and 2.0 and 8.2 months in the control cohort, respectively.

### 3.3. Association between Serum Factor and Clinical Outcomes

**Figure 3:**
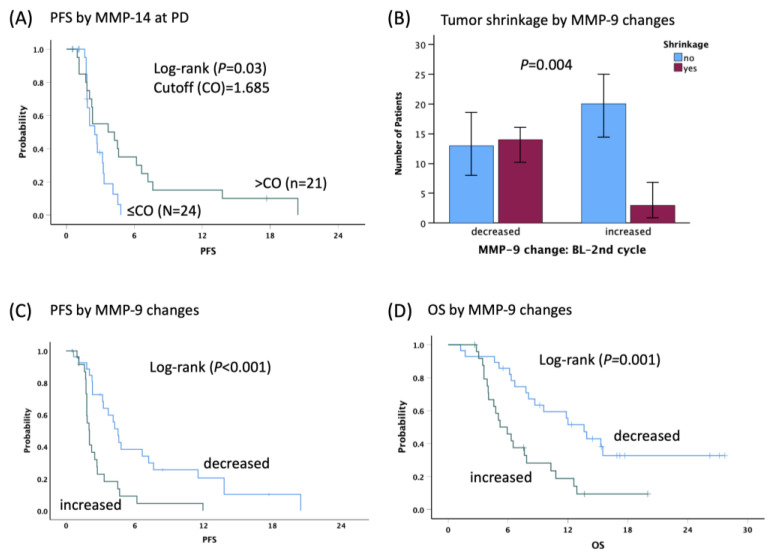
Progression-free survival (PFS) by MMP-14 levels using cutoff (CO) for tumor shrinkage at PD (**A**), tumor shrinkage (**B**), PFS (**C**), and overall survival (OS) (**D**) by MMP-9 change patterns (decreased vs. increased) between baseline (BL) and the second treatment cycle in the discovery cohort.

In the discovery cohort, high MMP-14 levels at baseline and PD were associated with TS (Table 2). To identify the optimal cut-off (CO) point that predicts TS at three points, the receiver operating characteristic curve (ROC) analysis was performed. High MMP-14 levels (>CO) at PD were associated with longer PFS (3.7 vs. 2.5 months, HR0.45, 95%CI: 0.21–0.95, *p* = 0.03) (Figure 3A) and correlated with TS at both BL (50% in >ROC vs. 19.2% in ≤ROC, *p* = 0.022) and PD (52.4% in >ROC vs. 9.5% in ≤ROC, *p* = 0.022).

Considering the correlation of MMP-14 expression with the regorafenib outcome, we further analyzed the relationship of regorafenib effect with other related factor expressions as well. We found that decreased changes in MMP-9 levels between BL and 2nd were associated with higher DCR (74% vs. 29%, *p* = 0.002) and TS (51.9% vs. 13%, *p* = 0.004), longer PFS (4.5 vs. 2.0 months, HR0.34, 95%CI: 0.18–0.65, *p* < 0.001), and OS (13.6 vs. 5.2 months, HR0.35, 95%CI: 0.18–0.68, *p* = 0.001) compared to increased changes (Figure 3B–D). In the control cohort, the findings observed in the discovery cohort were not as clear; however, 15 of 16 patients (93.8%) had decreased MMP-9 levels at 2nd (Supplementary Table S1).

### 3.4. Correlations among Serum Factors

**Figure 4:**
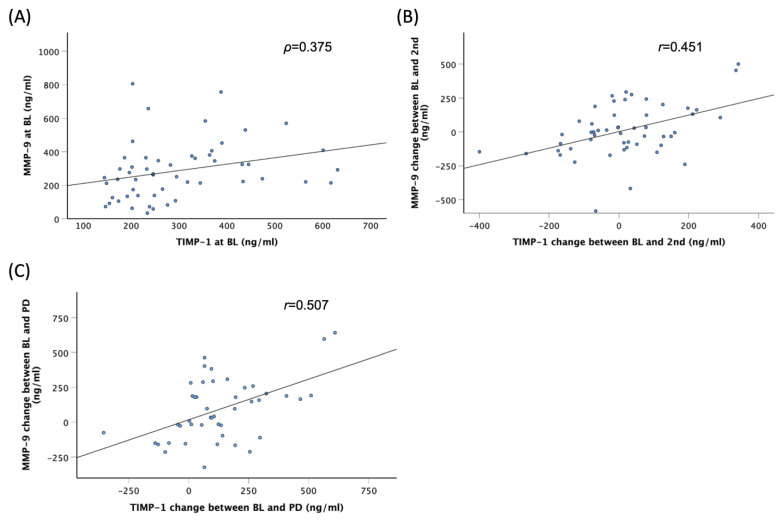
Correlation between serum MMP-9 and TIMP-1 levels at baseline (BL) (**A**), change in MMP-9 and TIMP-1 between BL and the second treatment cycle (2nd) (**B**), and change in MMP-9 and TIMP-1 between BL and progressive disease (PD) (**C**) in the discovery cohort.

In the discovery cohort, Spearman’s rank correlation coefficients between two serum factors at BL, 2nd, and PD, as well as their changes from BL to 2nd and PD, are shown in Supplementary Table S2. Moderate correlations were observed in the pair of MMP-9 and TIMP-1 at BL (*p* = 0.375) (Figure 4A). Additionally, moderate correlations were found in the pair of MMP-9 and TIMP-1 for changes between BL and 2nd (*r* = 0.451) and PD (*r* = 0.507) (Figure 4B,C). There were no significant correlations between other pairs.

## 4. Discussion

In this study, our aim was to explore candidate biomarkers of efficacy for regorafenib in patients with mCRC through preclinical omics analysis and translational validation studies. To our knowledge, we are the first to suggest the potential involvement of MMP-14 and MMP-9 in the mechanism of action of regorafenib, as evidenced by longitudinal monitoring of serum factor levels throughout the treatment.

Identification of biomarkers for novel agents that highly correspond to clinical outcome is difficult to detect in oncology practice. In the previous translational research targeting the angiogenic factors, baseline serum C–C motif chemokine ligand 5 (CCL5) levels and decreased serum vascular endothelial growth factor-A (VEGF-A) levels after initiation of treatment predicted the efficacy of regorafenib in refractory mCRC [14]. CCL5 exhibits late expression following T cell activation and localizes with tumor-infiltrating leukocytes [15,16]. The CCL5/CCR5 axis plays a role in the immune microenvironment and is exploited to facilitate tumor progression [17,18]. CCL3 and CCL4 are primarily produced by macrophages, dendritic cells, and lymphocytes, which activate CCR5 signaling downstream [19,20]. We previously reported the results of a unique pharmacogenetic study demonstrating that genetic variants in *CCL3*, *CCL4*, *CCL5*, and *CCR5* within the CCL5/CCR5 pathway predict the efficacy of regorafenib in mCRC [21]. Furthermore, the genetic functionality and biological roles of *CCL4* and *CCL3* genotypes were elucidated within the CCR5 network in patients with refractory mCRC treated with regorafenib [22]. According to our series of research studies on regorafenib, *CCL5* SNPs may impact not only CCL5-VEGF-A signaling via EPC but also the responses involving CCL3 and CCL4.

The current study employed a unique approach distinct from our previous research, combining preclinical omics analysis with translational validation to identify candidate biomarkers for regorafenib efficacy in patients with mCRC. Candidate biomarkers identified through preclinical research were subsequently validated using clinical samples. Utilizing a cancer cell panel informatics approach [13], we identified candidate biomarkers associated with regorafenib sensitivity. Specifically, an elevated level of MMP-14 was observed in a sensitive colorectal cancer cell line, whereas no significant changes were noted in the resistant cell line. Clinical data have already confirmed that soluble factors MMP-14 and MMP-9 play roles in the effectiveness of regorafenib in relation to cancer progression and metastasis [4,23]. MMP-14, in turn, degrades the extracellular matrix (ECM) and facilitates tumor invasion and angiogenesis [6,7]. Similarly, MMP-9, a gelatinase, contributes to ECM remodeling and has been implicated in the angiogenic switch during carcinogenesis [10]. Regorafenib targets several signaling pathways involved in tumor growth and angiogenesis, including the VEGF, PDGF, and RAF pathways [24]. Our findings suggest that the alteration of MMP-14 and MMP-9 levels by regorafenib may affect ECM degradation and angiogenesis, thereby suppressing tumor progression. TIMP-1 is a natural inhibitor of MMPs, such as MMP-2 and MMP-9 [12,25]. By binding to these MMPs, TIMP-1 inhibits their proteolytic activity, thereby reducing ECM degradation and potentially hindering cancer cell invasion and metastasis. Additionally, TIMP-1 also exhibits anti-apoptotic properties, which can contribute to tumor cell survival under certain conditions. This dual role makes TIMP-1 a complex and multifaceted regulator in the tumor microenvironment. The balance between MMPs and TIMPs is critical in determining the overall impact on tumor progression [26]. Additionally, by affecting the expression or activity of TIMP-1, regorafenib might influence the balance between MMPs and TIMPs, potentially enhancing its anti-tumor effects.

In our study, we observed an inverse correlation between MMP-14 and MMP-9 levels among mCRC patients treated with regorafenib, with higher levels of MMP-14 associated with a better prognosis in these patients. This finding may seem contradictory, as MMPs are typically associated with tumor invasion and metastasis. However, MMP-14 has complex roles that may vary based on the tumor context [6]. MMP-14, or MT1-MMP, is known to degrade extracellular matrix components and activate other MMPs, facilitating tumor invasion. However, recent studies have suggested that MMP-14 may also contribute to normalizing tumor vasculature, improving drug delivery and reducing tumor aggressiveness induced by hypoxia. Additionally, MMP-14 may enhance anti-tumor immunity by modulating immune cell activity within the tumor microenvironment [27]. Understanding these mechanisms might enable the development of therapies that leverage the benefits of MMP-14 while minimizing its potential for metastasis.

This study has several limitations. First, the sample size was limited in size, which may limit the generalizability of our findings. While our primary focus was identifying biomarkers for regorafenib efficacy, the limited size of the control group may have affected the robustness of our comparative analysis. Second, the observational design restricts our ability to establish causality between regorafenib treatment and changes in MMPs levels. Third, patient variability and prior treatments could have introduced confounding factors. Fourth, we did not assess other potential biomarkers or the long-term safety of regorafenib. Finally, we did not conduct loss-of-function studies, such as knockout experiments, for MMP-14 and MMP-9, which are important for validating their roles in regorafenib efficacy. Future larger, prospective studies are needed to validate these findings and address these limitations.

## 5. Conclusions

The regulation of MMP-14, MMP-9, and TIMP-1 by regorafenib reveals a complex interplay that affect therapeutic outcomes in patients with mCRC. Further studies are necessary to fully elucidate the precise mechanisms and explore potential combination therapies that could enhance the efficacy of regorafenib, such as combining it with antibodies against MMP-9 like andecaliximab [28,29] or FTD/TPI. For the clinical application of biomarkers, it is essential to validate them through robust preclinical data, as demonstrated in this study.

## Figures and Tables

**Table 1:** Baseline patients and tumor characteristics.

Cohort	Discovery Cohort (*n* = 53)		Control Cohort (*n* = 16)		
	*N*	%	*N*	%	*p*-Value
Sex					**0.016**
Male	28	52.8	3	18.8	
Female	25	47.2	13	81.3	
Age (year)					0.41
Median (range)	65 (34–78)		61 (45–77)		
≤65	26	49.1	10	62.5	
>65	27	50.9	6	37.5	
ECOG Performance status					0.99
ECOG 0	33	62.3	10	62.5	
ECOG 1	20	37.7	6	37.5	
Primary tumor site					**0.019**
Right	37	69.8	6	37.5	
Left	16	30.2	10	62.5	
Liver metastasis					0.42
Yes	34	64.2	12	75	
No	19	35.8	4	25	
Lung metastasis					**0.042**
Yes	28	52.8	13	81.3	
No	25	47.2	3	18.8	
Lymph node metastasis					0.582
Yes	29	54.7	10	62.5	
No	24	45.3	6	37.5	
Peritoneal metastasis					1.0
Yes	12	22.6	4	25	
No	41	77.4	12	75	
Number of metastases					0.052
<2	18	34.0	1	6.3	
≥2	35	66.0	15	93.8	
Primary tumor resected					1.0
Yes	44	83.0	14	87.5	
No	9	17.0	2	12.5	
Adjuvant history					0.145
Yes	19	35.8	9	56.3	
No	34	64.2	7	43.8	

ECOG: Eastern Cooperative Oncology Group. *p* value was based on Chi-square test or the Mann–Whitney test when appropriate. *p*-values < 0.05 are shown in bold.

**Table 2:** Serum MMPs levels in discovery.

MMPs	Point	Non-TS (*n* = 33)	TS (*n* = 17)	*p*-Value *	Non-DC (*n* = 24)	DC (*n* = 27)	*p*-Value *
MMP-9,	BL	272.58 ± 172.63	329.29 ± 177.06	0.28	250.86 ± 131.02	330.33 ± 197.98	0.10
(mean ± SD, ng/mL)	2nd	345.05 ± 217.60	231.21 ± 113.50	**0.019**	352.49 ± 192.27	267.32 ± 188.83	0.12
	PD	405.66 ± 228.78	354.82 ± 224.69	0.51	400.84 ± 172.13	377.93 ± 267.04	0.74
MMP-9,	BL–2nd	72.47 ± 177.013	−98.07 ± 193.79	**0.003**	101.63 ± 178.81	−63.01 ± 180.82	**0.002**
(mean ± SD, ng/mL)	BL–PD	130.24 ± 209.37	29.32 ± 250.31	0.18	152.01 ± 96.70	44.32 ± 237.07	0.11
MMP-14,	BL	1.91 ± 0.81	2.66 ± 1.01	**0.006**	2.54 ± 2.37	2.23 ± 1.08	0.55
(mean ± SD, ng/mL)	2nd	2.03 ± 0.99	2.42 ± 0.73	0.16	2.02 ± 1.05	2.30 ± 0.076	0.29
	PD	1.71 ± 0.86	2.34 ± 0.57	**0.02**	1.71 ± 0.92	2.06 ± 0.72	0.17
MMP-14,	BL–2nd	0.15 ± 0.78	−0.23 ± 1.24	0.19	−0.52 ± 2.31	0.09 ± 1.19	0.25
(mean ± SD, ng/mL)	BL–PD	−0.04 ± 0.64	−0.24 ± 1.0	0.44	−0.75 ± 2.62	−0.02 ± 0.84	0.22
MMP-2,	BL	345.10 ± 142.75	354.43 ± 114.68	0.82	341.49 ± 143.82	359.11 ± 124.09	0.64
(mean ± SD, ng/mL)	2nd	277.79 ± 138.51	289.11 ± 100.37	0.77	273.89 ± 117.14	296.44 ± 137.49	0.54
	PD	334.86 ± 120.84	295.05 ± 127.09	0.34	320.36 ± 96.14	327.38 ± 143.95	0.85
MMP-2,	BL–2nd	−72.08 ± 144.68	−65.32 ± 124.85	0.87	−67.60 ± 133.50	−69.08 ± 140.50	0.97
(mean ± SD, ng/mL)	BL–PD	−23.00 ± 134.42	−45.80 ± 105.37	0.59	−31.22 ± 121.08	−31.87 ± 129.95	0.99
TIMP-1,	BL	307.79 ± 122.57	287.63 ± 151.96	0.61	303.80 ± 125.73	296.75 ± 137.62	0.85
(mean ± SD, ng/mL)	2nd	358.80157.13300	239.22 ± 81.43	**0.001**	380.85 ± 164.64	254.98 ± 93.39	**0.002**
	PD	464.90194.60728	362.91 ± 292.72	0.19	483.44 ± 218.41	377.60 ± 232.69	0.13
TIMP-1,	BL–2nd	48.84 ± 146.24	−48.40 ± 127.21	**0.025**	77.05 ± 139.07	−44.01 ± 126.74	**0.002**
(mean ± SD, ng/mL)	BL–PD	171.08 ± 167.43	54.43 ± 232.49	0.07	197.76 ± 178.05	69.04 ± 190.33	**0.027**

MMPs, matrix metalloproteinases; TIMP, tissue inhibitor of metalloproteinase; BL, baseline; 2nd, before second cycle; PD, progressive disease; TS, tumor shrinkage; DC, disease control; SD, standard deviation. Differences in the mean MMPs levels were tested using Student’s unpaired *t* test. * *p*-values < 0.05 are shown in bold.

## Data Availability

The data presented in this study are available on request from the corresponding author.

## Supplementary Materials

The following supporting information can be downloaded at: https://www.mdpi.com/article/10.3390/cancers16162855/s1, Figure S1: Role of MMPs and TIMPs; Table S1: Serum MMPs levels in control cohorts; Table S2: Correlations among serum factors.
