# Supplementary material for: Exploratory Study Identifies Matrix Metalloproteinase-14 and -9 as Potential Biomarkers of Regorafenib Efficacy in Metastatic Colorectal Cancer
Source: Cancers (Basel). 2024 Aug 15;16(16):2855. doi: 10.3390/cancers16162855 (PMC11352555; doi:10.3390/cancers16162855)
Supplement: Supplementary file 1 [file cancers-16-02855-s001.zip › Table S1.pdf]

Supplementary Table S1. Serum MMPs levels in control cohorts

MMPs	Point	Non-TS (n=11)	TS (n=5)	*P*-value*	Non-DC (n=11)	DC (n=)	*P*-value*
MMP-9	BL	316.47±164.67	305.57±107.82	0.90	336.97±158.29	260.46±108.84	0.35
(mean ± SD, ng/ml)	2nd	116.59±112.33	123.62±157.22	0.92	117.24±111.98	122.19±157.91	0.94
	BL–2nd	-199.88±118.73	-181.95±150.73	0.80	-219.73±120.37	-138.27±127.73	0.24
MMP-14	BL	1377.33±326.77	1244.50±286.91	0.45	1414.81±319.60	1162.04±232.73	0.14
(mean ± SD, ng/ml)	2nd	1471.149±298.70	1143.14±252.79	0.052	1483.84±289.88	1115.23±229.29	**0.026**
	BL–2nd	93.82±247.86	-101.36±240.03	0.163	69.03±261.74	-46.82±247.24	0.42

BL, baseline; 2nd, before second cycle; TS, tumor shrinkage; SD, standard deviation

Differences in the mean MMPs levels were tested using Student's unpaired *t* test.

**P*-values <0.05 were shown in bold.
