# Supplementary material for: Exploratory Study Identifies Matrix Metalloproteinase-14 and -9 as Potential Biomarkers of Regorafenib Efficacy in Metastatic Colorectal Cancer
Source: Cancers (Basel). 2024 Aug 15;16(16):2855. doi: 10.3390/cancers16162855 (PMC11352555; doi:10.3390/cancers16162855)
Supplement: Supplementary file 1 [file cancers-16-02855-s001.zip › Table S2.pdf]

Supplementary Table S2. Correlations among serum factors

Pairs of factors	TIMP-1–MMP-9	TIMP-1–MMP-2	TIMP-1–MMP-14	MMP-9–MMP-2	MMP-9–MMP-14	MMP-2–MMP-14
Points						
BL	ρ=0.375*	ρ=0.177	ρ=−0.044	ρ=0.188	ρ=−0.001	ρ=−0.027
2nd	ρ=0.263	ρ=−0.095	ρ=−0.216	ρ=−0.112	ρ=−0.229	r=0.146
PD	ρ=0.229	ρ=0.174	ρ=–0.15	ρ=0.069	ρ=−0.091	ρ=−0.145
Changes						
BL–2nd	r=0.451*	r=0.08	ρ=–0.1	ρ=−0.036	r=−0.129	ρ=0.024
BL–PD	r=0.507*	r=0.296	ρ=0.096	r=0.093	ρ=−0.038	ρ=–0.031

BL, baseline; 2nd, before second cycle; PD, progressive disease

Correlations were estimated using Spearman's rank correlation coefficient (ρ) or Pearson's correlation coefficient (r) according to data distribution.

**P* values were <0.05.
