# Supplementary figures and images for: Exploratory Study Identifies Matrix Metalloproteinase-14 and -9 as Potential Biomarkers of Regorafenib Efficacy in Metastatic Colorectal Cancer
Source: Cancers (Basel). 2024 Aug 15;16(16):2855. doi: 10.3390/cancers16162855 (PMC11352555; doi:10.3390/cancers16162855)

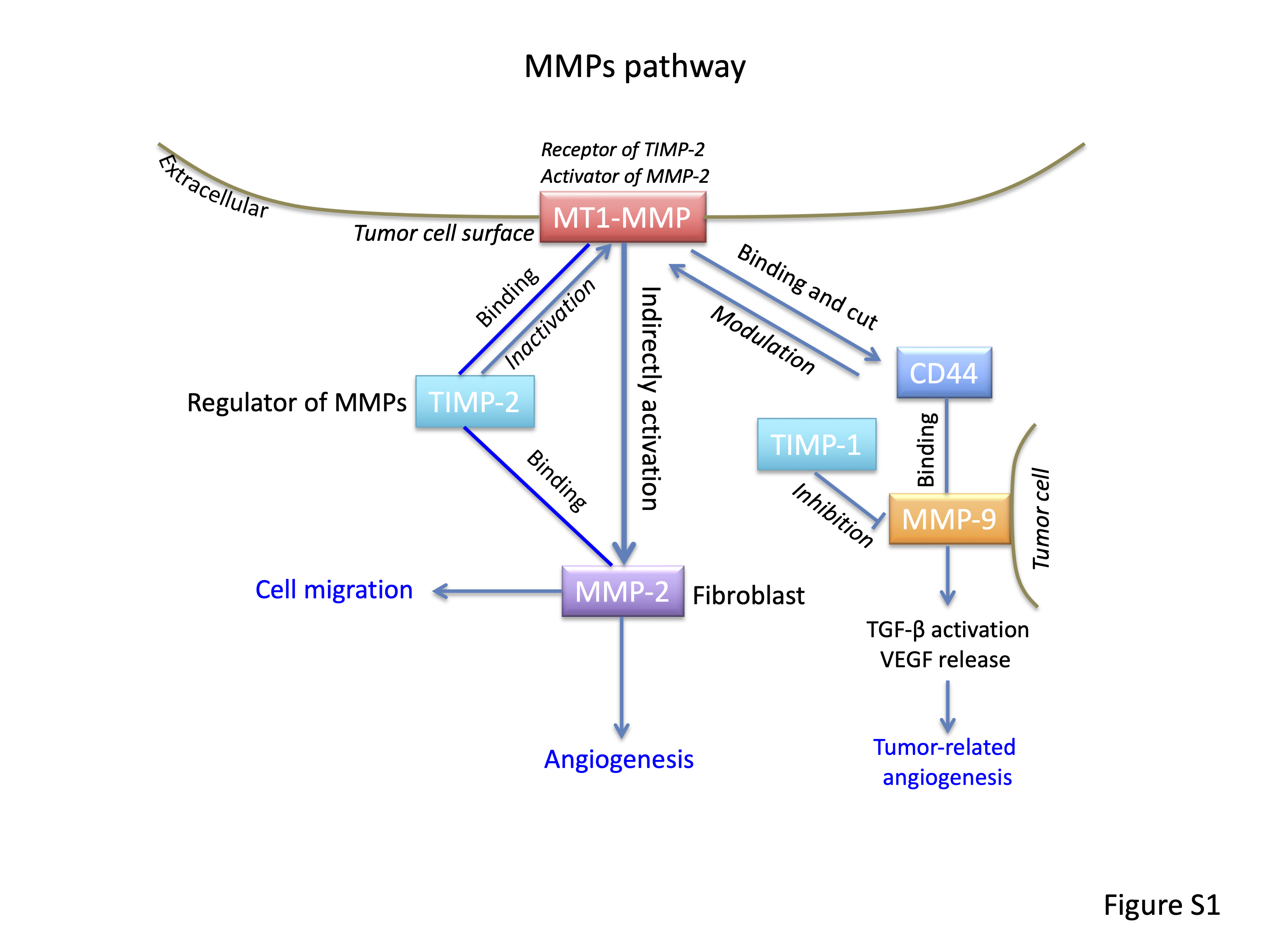

Supplement: Supplementary file 1 [file cancers-16-02855-s001.zip › Figure S1.tiff]
